# P-763. Assessment of the *in vitro* potency of anti-mycobacterial agents against clinical *Mycobacterium avium* complex (MAC) pulmonary isolates: implications of intravenous and inhaled amikacin breakpoint reporting

**DOI:** 10.1093/ofid/ofae631.958

**Published:** 2025-01-29

**Authors:** Christian Mark Gill, Robin R Chamberland, Getahun Abate

**Affiliations:** SSM Health Saint Louis University Hospital, St. Louis, Missouri; Saint Louis University School of Medicine, St. Louis, Missouri; Saint Louis University, St. Louis, MO

## Abstract

**Background:**

MAC pulmonary disease is associated with significant morbidity and requires multi-drug treatment for extended periods. The novel dosage form amikacin liposome inhalation suspension (ALIS) represents a much-needed treatment option and is noted to have success at MICs of ≤64mg/L, higher than the intravenous (IV) amikacin (AMK) breakpoint (≤16 mg/L) due to optimized pulmonary exposure. Herein, we assessed the *in vitro* potency of AMK utilizing both ALIS and IV breakpoints as well as other standard antimycobacterial agents.

**Methods:**

MAC isolates obtained from sputum, bronchoalveolar lavage, or lung tissue culture between 1/1/2018 - 3/1/24 were assessed. The first isolate per patient per year was assessed. MICs were collected from the medical record as determined by standard methods at one of two reference laboratories. If a single sample had two MIC values reported, the more conservative was used for analysis. MICs to AMK, clarithromycin (CLR), moxifloxacin (MXF), and linezolid (LNZ) were collected. Data were assessed as MIC50/90 (mg/L) and categorized per CLSI breakpoints. Patients were classified as receiving MAC directed treatment based on documentation in the medical record. If treatment data was unknown, patients were classified as indeterminate treatment.

**Results:**

218 samples were included from 178 patients. CLR was the most active agent with 97% of isolates testing as susceptible (MIC50/90, 0.5/2). MXF and LNZ had relatively poor activity with 82% and 66% of isolates susceptible to each agent, respectively. Considering AMK, high susceptibility to the IV breakpoint was observed with 94% of isolates meeting this criterion (MIC50/90, 8/16). Among the 14 isolates that were intermediate or resistant to IV AMK, 13 remained susceptible to ALIS (MIC range 32-64). 54%, 26%, and 20% of patients were treated, untreated, or indeterminate, respectively. The percent of isolates susceptible to each agent between treated and untreated were similar for all agents.

**Conclusion:**

AMK and CLR remain highly active against MAC pulmonary isolates. Amongst isolates intermediate or resistant to IV AMK, most isolates remained susceptible to ALIS. These data highlight the need for laboratories to report susceptibility to both IV and ALIS to avoid clinicians erroneously disregarding a therapeutic option.

**Disclosures:**

**Christian Mark Gill, PharmD, BCIDP**, Cepheid: Grant/Research Support|Entasis: Grant/Research Support|Everest Medicines: Grant/Research Support|Shionogi: Grant/Research Support

